# Some Causes of the Variable Shape of Flocks of Birds

**DOI:** 10.1371/journal.pone.0022479

**Published:** 2011-08-04

**Authors:** Charlotte K. Hemelrijk, Hanno Hildenbrandt

**Affiliations:** Behavioural Ecology and Self-Organisation, Centre for Ecological and Evolutionary Studies, University of Groningen, Groningen, The Netherlands; German Cancer Research Center, Germany

## Abstract

Flocks of birds are highly variable in shape in all contexts (while travelling, avoiding predation, wheeling above the roost). Particularly amazing in this respect are the aerial displays of huge flocks of starlings (*Sturnus vulgaris*) above the sleeping site at dawn. The causes of this variability are hardly known, however. Here we hypothesise that variability of shape increases when there are larger local differences in movement behaviour in the flock. We investigate this hypothesis with the help of a model of the self-organisation of travelling groups, called StarDisplay, since such a model has also increased our understanding of what causes the oblong shape of schools of fish. The flocking patterns in the model prove to resemble those of real birds, in particular of starlings and rock doves. As to shape, we measure the relative proportions of the flock in several ways, which either depend on the direction of movement or do not. We confirm that flock shape is usually more variable when local differences in movement in the flock are larger. This happens when a) flock size is larger, b) interacting partners are fewer, c) the flock turnings are stronger, and d) individuals roll into the turn. In contrast to our expectations, when variability of speed in the flock is higher, flock shape and the positions of members in the flock are more static. We explain this and indicate the adaptive value of low variability of speed and spatial restriction of interaction and develop testable hypotheses.

## Introduction

The beautiful coordination in flocks of birds has raised scientific interest since ages in both laymen and scientists [1], [2], [3], [4], [5]. Flocks of birds have great variation in shape: often different flocks have different shapes and a single flock changes its shape over time [1], [5], [6]. Extreme changes in shape and density of flocks occur during the aerial displays of thousands of starlings at dusk. For instance, sometimes during turning the flock may change in relative proportions, density and volume [7], [8], whereas at other times the shape of a flock may remain intact while only changing its orientation relative to the movement direction [4]. Further, during turning individuals may reposition their location within a flock in an amazing way [1], [4], [5], [8]. This variability of shape differs markedly from what is described for schools of fish. Schools of fish are usually oblong in the movement direction [9], [10], [11]. However, under specific conditions, shapes of schools of fish are variable too, for instance, when a school is very large, and also when it is attacked by a predator. Very large schools have been described to be amorphous and to comprise extensions at the border, so-called pseudopodia, and sparse areas in the interior, called vacuoles, as if they consist of subgroups that move in somewhat different directions [12]. Similarly, in our model of very large schools (comprising up till 10.000 individuals) in which individuals have a limited view because it is blocked by those that are closest around them, shape appears more variable than in other models. This is due to the occurrence of subgroups with different movement directions in the school (Kunz and Hemelrijk, under review). Further, when being under attack of a predator, the shape of schools may become highly diverse. The shapes that emerge are for instance coined as ‘bend’, ‘flash expansion’, ‘herd’, ‘split’, and ‘hour glass’ [13]. Computer models of such attacks show that this diversity arises from the local differences of prey behaviour in the flock [14], [15]. These depend on the prey's distance to the predator: Individuals close to the predator are avoiding it, while those further away from the predator are coordinating with the other school members. In conclusion it seems that the variability of school shape may arise from local differences in movement behaviour, thus, from reduced synchronisation of the school of fish.

Since it is very difficult to study empirically [16] whether local differences in behaviour lead to a greater variation of shapes of flocks of birds, we will study it in a model of self-organised travelling groups, because such models have helped to create a better understanding of travelling groups in many aspects, such as their alignment [17], [18], [19] and direction choice [20], [21] and, most importantly, also their shape. They show, for instance, that shape of a group of fish and birds changes when it is under attack of a predator [14], [15], [22], that shape of fish schools depends on the synchronisation of spawning tendency [23], and on density and school size [24], [25], [26].

Our models of fish schools have shown that the commonly observed oblong shape in the movement direction emerges as a side-effect of coordination and slowing down to avoid collisions [24], . The elongated shape emerges, for all school sizes, in models in two dimensions or three, when individuals move at slow speed or fast and when a single school comprises individuals of a single body size or of two sizes. Furthermore, in our models of fish schools, schools appear to be more oblong the greater the number of individuals they include. We have confirmed these patterns in our empirical studies of three-dimensional positions of individuals in schools of 10 to 60 mullets: larger schools are both, denser and more oblong [25]. We attribute the fact that larger schools are more oblong to the higher number of adjustments necessary to avoid collisions in larger schools, because in larger schools individuals are closer to their nearest neighbours up till a certain saturation point[24]. Individual fish in larger schools are closer to their nearest neighbours. This emerges, because the attraction to other school members in larger schools is stronger because of the higher number of interaction partners.

In our model of bird flocks [26], however, like in flocks of real birds [4], the relationship between density and group size is known to be absent. This may be due to the usually much larger group sizes that are investigated in studies of flocks of birds than of schools of fish: The flock sizes studied are already in the range in which density is saturated.

In our present study of bird flocks we will use a model, called StarDisplay [26]. StarDisplay combines an adapted version of our former model of travelling schools of fish with characteristics of birds [24], [26]. Modelled individuals fly following simplified aerodynamics, i.e. they experience lift, drag and the force of gravity [29] and in order to fly along a curve, like real birds, individuals roll into the direction of the turn until they are at a certain angle to the horizontal plane, the so-called banking angle [30]. The model is parameterised so that individuals resemble starlings, as regards body weight, speed, lift-drag coefficient [31], roll rate [26], number of interaction partners [4] and the way in which the flocks remain above a sleeping site of size similar to that of Termini in Rome [7], [32].

Its patterns of flocking have been shown to resemble remarkably those of huge flocks of real starlings when flying above the roost recently studied with the help of stereo-photography above Rome [4]. The resemblance concerns the flat shape of flocks, the relative proportions (aspect ratios) of the flock shapes, their distribution of distances and angles to the nearest neighbours, their orientation, their balanced density between front and back and the way flocks turn [26].

Here, we investigate to what degree flock shape and its variability depends on local differences in behaviour. We assume that greater local differences in behaviour arise from larger flock size, lower number of interaction partners, sharp turning, rolling during turning and greater adjustment (and thus variability) of speed. We confirm that these traits cause larger differences in behaviour among individuals that indeed result in a greater variability of shape, except for one trait, namely variability of speed. We explain how high variability of speed results in low variability of shape of the flock. We derive testable hypotheses for real animals and speculate about the adaptive value of locality of interaction and adjustment of speed.

## Methods

### The Model

The behaviour of each individual in StarDisplay is based on its cruise speed, its social environment (i.e. the position and heading of its nearby neighbours), its attraction to the roost and the simplified aerodynamics of flight which includes banking while turning [26]. Following other studies [2], [24], [33], we model social coordination in terms of (social) forces. Because flying implies movement in all directions, our model is three dimensional. We built the model in SI units and choose real parameter values where available (Tab. 1).

### Details of behavioural rules

**Figure 1:**
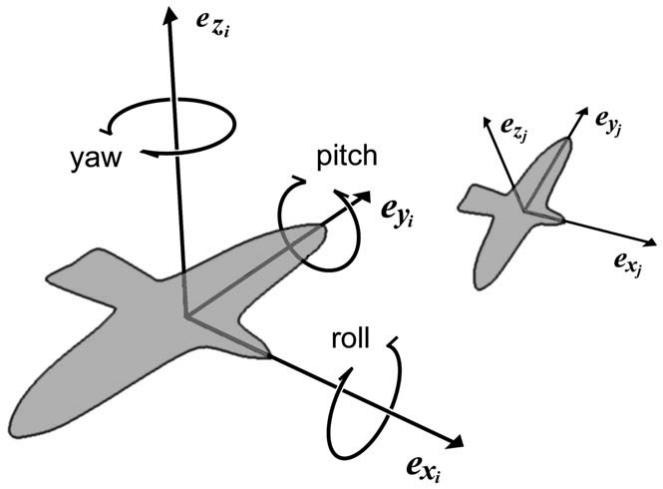
Local co-ordinate system. The local co-ordinate systems of 2 birds with different orientations in space and at different distances to the viewer. 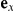 is the bird's forward direction; 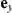, its sideways direction; and 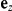, its upward direction. It can change these by rotating around these 3 principal axes (roll, pitch and yaw).

Each individual is characterised by its mass, 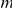, its speed, 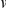, and its location, 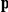. Its orientation in space is given by its local coordinate system 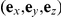. Following the model by Reynolds [2], its orientation is indicated by its forward direction, 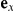, its sideward direction, 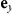, and its upward direction, 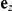, which it changes by rotating around these three principal axes (*roll*, *pitch* and *yaw*) (Fig. 1).

As to its speed, a force, 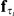 (Equ. 1) brings an individual back to its cruise speed 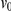 after it has deviated from it [24].?up?>
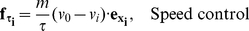
(1)where 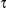 represents the relaxation time, 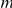 is the mass of the individual 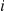 and 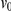 its cruise speed, 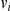 is its speed, and 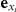 its forward direction.

**Figure 2:**
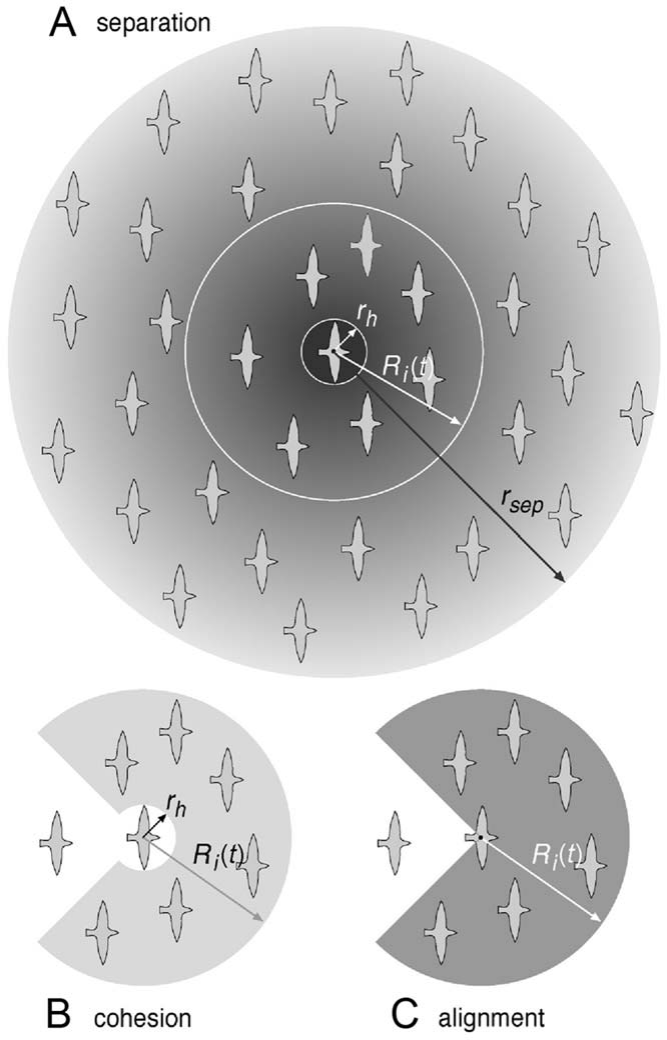
Social interaction. Social interaction ranges for separation (A), cohesion (B), and alignment (C). Note that the lengths of the different radii in the figure are not to scale with the default values in Table 1.

To make each individual interact with a specific constant number of its closest neighbours (i.e. topological range), each individual 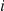 in the model adjusts its metric interaction range, 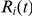 [24] following Equ. 2,3.
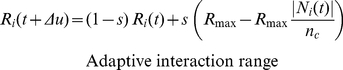
(2)

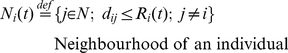
(3)where 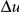 is the reaction time, 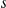 is an interpolation factor, 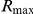 is the maximal metric interaction range, 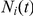 is the neighbourhood of individual 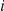 at time 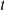, i.e. the set of neighbours of an individual 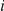 which is composed of 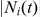 neighbours from the total flock, 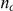 is the fixed number of topological interaction partners it strives to have and 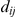 is the distance between individual 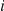 and 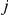 given by 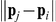 where 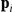 gives the position of an individual 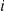. Thus, the radius of interaction at the next step in reaction-time, 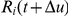, increases whenever the number of interaction partners 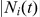 is smaller than the targeted number 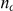, and it is decreased if it is larger than that; it remains as before if 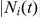 equals 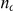. Here 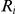 can neither decrease below the hard sphere in which individuals are maximally avoiding each other 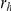 (Equ. 4, Fig. 2) nor increase beyond 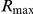. 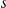, the interpolation factor, determines the step-size of the changes and thus, the variance of the number of actual influential neighbours.

As to separation, individual 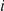 is led by a force 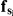 to move in the opposite direction of the average direction of the locations of the 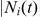 others in its neighbourhood (Fig. 2). Following others [14], [33], we have omitted the blind angle at the back (Equ. 4 and see Parameterization & Experiments). We gave individuals a hard sphere with radius 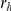 as mentioned above, in which they avoid each other maximally (Equ. 4). Outside the hard sphere, but inside the radius of separation 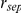, the degree of avoidance of others decreases with the distance to the neighbour following a halved Gaussian, 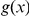, with 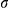 the standard deviation of the Gaussian set so that at the border of the separation zone the force is almost zero, 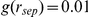 (Equ. 4).
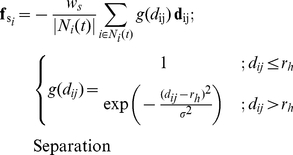
(4)Here, 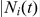 is the number of individuals in the neighbourhood of interaction (Equ. 3) and 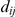 is the distance from individual 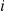 to individual 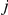. The direction from individual 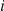 to individual 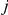 is specified by the unit vector 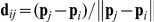 and 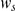 is the weighting factor for separation (Tab. 1).

As to cohesion, individual 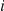 is attracted by a force 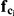 to the direction of the centre of mass (i.e. the average *x*, *y*, *z* position) of the group of 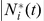 individuals located in its topological neighbourhood, but not in its blind angle, in a way similar to models of others [27], [28], [33], [34], [35]. Here, 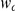 is the weighing factor for cohesion (Equ. 5, Tab. 1). Within the radius of the hard sphere 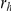, we ignore cohesion with others (Equ. 5). To represent fear of predators [36] and build a sharp boundary of the flock [4], we make individuals cohere more strongly when they are at the border of the flock than in its interior by multiplying the force of cohesion by a factor indicating the degree to which an individual is peripheral (Equ. 5, 7). This factor, called ‘centrality’ in the group, 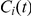, we calculate as the length of the average vector of the direction of all its neighbours 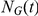 relative to the individual 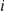, [37]. A high value indicates that the individual is peripheral; a lower value indicates that it is located more in the centre of the group. The ‘neighbouring’ individuals are all 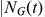 individuals in a radius of twice the actual perceptual distance of the individual 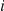 (Equ. 7).
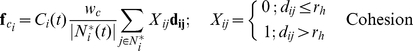
(5)

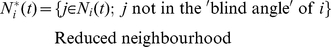
(6)

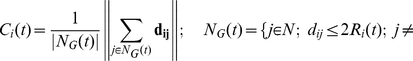
(7)


As regards its alignment behaviour (Equ. 8), individual 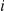 feels a force, 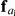, to align with the average forward direction of its 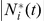 interaction neighbours (the same neighbours as to whom it is attracted, Fig. 2BC).
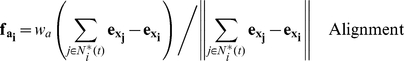
(8)Here, 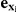 and 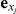 are the vectors indicating the forward direction of individuals direction of individuals 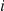 and 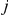 and 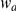 is the fixed weighting factor for alignment (Tab. 1).

**Figure 3:**
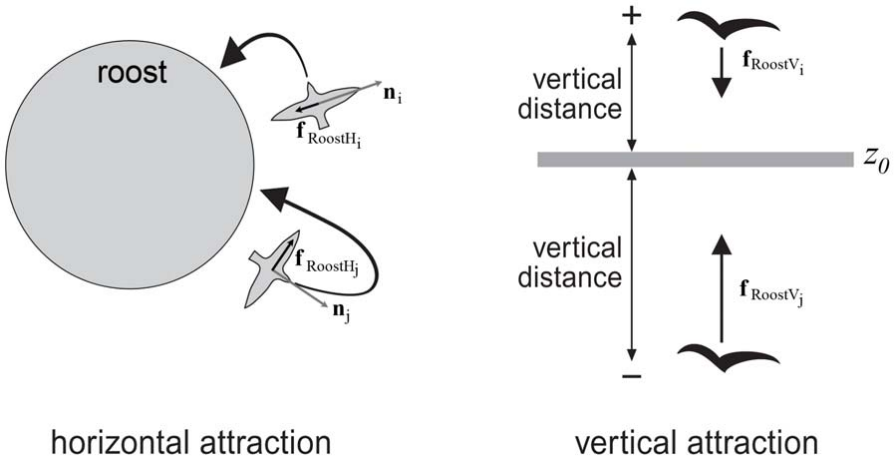
The roost. Pictorial representation of attraction to the roost. A) horizontal attraction 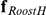, normal 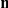 and intended trajectory for individuals *i* and *j*. Trajectories indicate that the turning is sharpest when flying out radially. B) vertical attraction 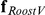.

The ‘social force’ is the sum of these three forces (Equ. 9).
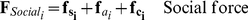
(9)Individuals fly at a similar height above the sleeping site like real starlings [7], because we made them experience both in a horizontal and vertical direction a force of attraction 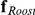 to the ‘roosting area’ (Equ. 10, 11, 12, Fig. 3). The strength of the horizontal attraction, 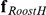, is greater, the more radially it moves away from the roost; it is weaker if it is already returning (Fig. 3A). The strength is calculated using the dot product, i.e. the angle between the forward direction of individual *i*, 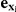, and the horizontal outward-pointing normal 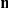 of the boundary. The range of the result [−1..1] is transformed to [0..1] by halving the dot product and summing it with a 1/2. The actual direction of the horizontal attraction force to the roost is given by 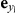 which is the individual's lateral direction. The sign in Equ. 11 is chosen to reduce the outward heading. The actual direction of the horizontal attraction force is given by 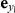 which is the individual's lateral direction. Vertical attraction, 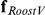, is proportional to the vertical distance from the preferred height 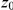 above the roost (arbitrarily called the zero level, Fig. 3B). Here 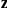 is the vertical unit vector. 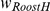 and 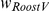 are fixed weighting factors.
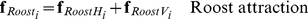
(10)

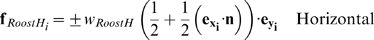
(11)

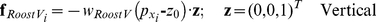
(12)


The random force indicates unspecified stochastic influences (Equ. 13) with 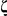 being a random unit vector from a uniform distribution and 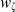 being a fixed scaling factor. The sum of the social force, the forces that control speed and ranging and the random force is labeled as ‘steering force’ (Equ. 14).
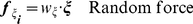
(13)

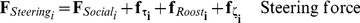
(14)


**Figure 4:**
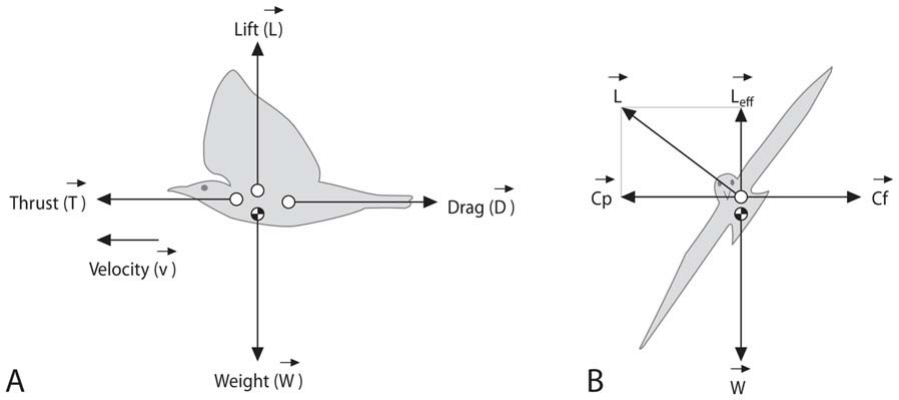
Aerodynamics. Aerodynamic forces, A) while flying straight and B) while banking. 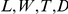: lift, weight, thrust, drag respectively. 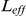 = effective lift, 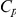 = centripetal force, 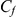 = centrifugal force.

Physics of flight in the model follows the standard equations of fixed wing aerodynamics which link the lift 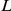, the drag 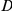 and the thrust 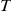 produced by a bird to attain its current speed 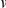 (Equ. 15a, Fig. 4):
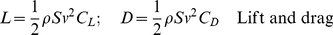
(15a)

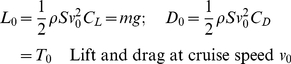
(15b)

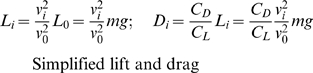
(15c)where 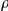 is the density of the air and 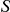 represents the wing area of the bird (of identical size for all birds). The quotient of 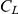 and 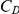 of the dimensionless lift and drag coefficients in the model is fixed, resembling the almost fixed ratio in reality [29]. When a bird is flying horizontally while maintaining a constant cruise speed 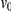 its lift balances its weight 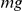 (mass times gravity) and its thrust balances its drag (Equ. 15b, Fig. 4A). Division of 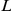 by 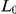 and of 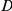 by 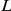 in Equ. 15ab yields Equ. 15c in which the lift and the drag only depend on the actual speed.

Gravity is directed towards the global ‘down’ direction, 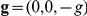, the lift upwards operates towards the local ‘up’ direction 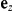 of the bird and the drag is pointing in the direction opposite to its actual ‘forward’ direction 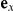 (Fig. 4). Thus, the flight forces are:
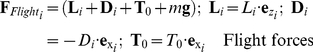
(16)Real birds roll into the turn in order to make turns [30]. Because in the absence of external influence we assume that birds ‘intend’ to fly with their wings at a horizontal level in order to move straightforward, we give the model-birds a tendency to roll back. To represent banked turns (Fig. 4B), we first calculate the degree to which individuals want to turn, i.e. their lateral acceleration, 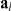, which is exerted by the steering force. Banking implies that the individual rolls around its forward axis in the direction of its lateral acceleration, 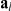. The lateral acceleration follows the first law of Newton (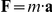),
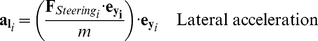
(17)

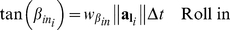
(18)

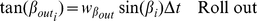
(19)

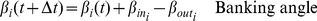
(20)where 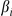 is the actual banking angle, 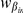 and 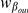, respectively are the weights for rolling in and out the curve of turning, 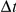 is the update time and 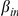 and 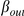 are the angles over which an individual intends to move inwards and outwards. The tendency to roll into the turn increases with the strength of the tendency to turn sideways, which is due to the urge to coordinate with its topological neighbours and to stay above the roost (Equ. 18, Fig. 4B). Once an individual has banked in the model, its tendency to roll back to the horizontal is proportional to its actual banking angle (Equ. 19). The actual banking angle (Equ. 20) is the sum of the current angle and the tendencies to roll-in and to roll-out. The ratio of 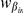 and 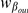 determines the roll rate. Note that by banking the individual creates a centripetal force at the cost of lift (Fig. 4B). Consequently it temporarily tends to move downwards.

After summing the forces of steering and flying, we use Euler integration to calculate the position and velocity at the end of each time-step 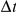:
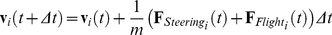
(21)

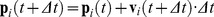
(22)where 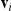 is the velocity of individual 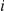, *m* its mass, 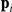 its location, and 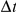 is the update time. For the default values, see table 1.

**Table 1:** Default parameter values^1^.

Parameter	Description	Default value
*Δt*	Integration time step	5 ms
*Δu*	Reaction time	50 ms [43]
*v_0_*	Cruise speed	10 m/s [31]
*M*	Mass	0.08 kg [31]
*C_L_/C_D_*	Lift-drag coefficient	3.3 [31]
*L_o_*	Default lift	0.78 N (Equ. 15)
*D_0_,T_0_*	Default drag, default thrust	0.24 N (Equ. 15)
*w_βin_*	Banking control	10 (starling videos)
*w_βout_*	Banking control	1 (starling videos)
*Τ*	Speed control	1 s
*R_max_*	Max. perception radius	100 m
*n_c_*	Topological range	6.5 [4]
*S*	Interpolation factor	0.1 *Δu*
*r_h_*	Radius of max. separation (“hard sphere”)	0.2 m [4]
*r_sep_*	Separation radius	4 m [after 4])
*Σ*	Parameter of the Gaussian *g(x)*	1.37 m [after4])
*w_s_*	Weighting factor separation force	1 N
	Rear “blind angle” cohesion & alignment	2*45°
*w_a_*	Weighting factor alignment force	0.5 N
*w_c_*	Weighting factor cohesion force	1 N
*C_c_*	Critical centrality below which an individual is assumed to be in the interior of a flock.	0.35
*w_ξ_*	Weighting factor random force	0.01 N
*R_Roost_*	Boundary radius	150 m [7]
*w_RoostH_*	Weighting factor horizontal boundary force	0.01 N/m (starling videos)
*w_RoostV_*	Weighting factor vertical boundary force	0.2 N [7]

1 Note that *D_0_* and *T_0_* are calculated by equation 16 by inserting *v_0_* for *v_i_*. For more details on parametrization, see our previous study 26. Hildenbrandt H, Carere C, Hemelrijk CK (2010) Self-organized aerial displays of thousands of starlings: a model. Behavioral Ecology 21: 1349–1359 doi:1310.1093/beheco/arq1149.

### Parameterization and Experiments

We have used the parameterization to realistic data of birds, especially of starlings, from our earlier version of StarDisplay (Tab. 1) [26]. To study the effects of locality of interaction we performed several experiments in the model [38], that concern 1) the group size, 2) the number of influential neighbours (i.e. topological range), 3) the attraction to the roost, 4) the banking during turning, and 5) the variability of speed.

### Measurements

We measured the following properties of a flock: its shape (relative proportions and orientation), its volume, the correlation length of the deviations from the average velocity among its group members, its polarization (global and local), and its average degree of banking.

In our timeseries, we represent each property averaged over all individuals of a flock, if appropriate. When characterising a flock of a certain size, we averaged values also over time (i.e. 30 minutes while measuring it once per second).

**Figure 5:**
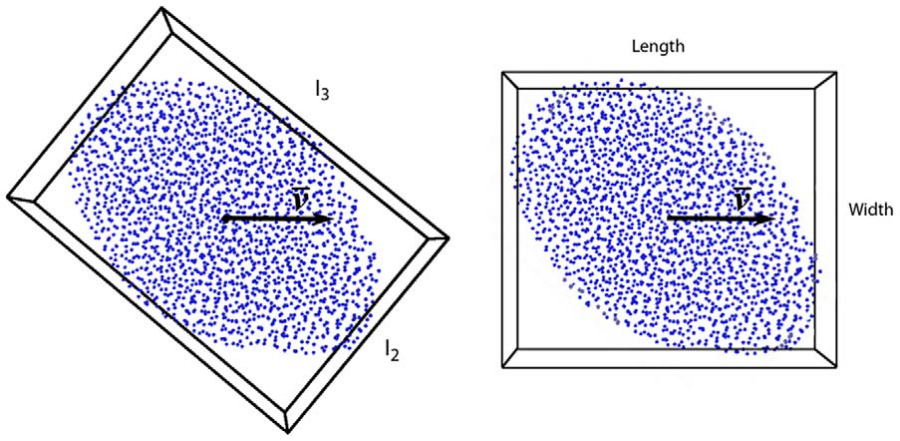
Measuring of shape. Two ways of measuring shape. Left: Measurement of 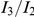 based on PCA analysis. Right: measuring of elongation, 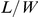, length in the movement direction and width orthogonally to it. Birds fly from left to right, flock is shown from above.

The flock shape we measure in several ways, namely by its relative dimensions in three aspect ratios, by the degree to which it is oblong as measured by the longest dimension over the medium one 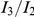 and by the degree to which it is elongated in the direction of movement, L/W (Fig. 5). We measure the relative dimensions of the flock by enclosing the flock in the bounding box of minimum-volume parallel to the longest dimension. This bounding box is calculated by means of a principal component analysis (PCA) of the coordinates of the flock members [39] (Movie S1). The eigenvectors that are associated with the smallest/medium/largest eigenvalue of the covariance matrix provide an orthonormal coordinate system oriented along the axes defining the shortest/medium/longest dimensions of the flock, respectively, 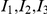. Note that the shortest dimension 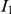 corresponds to the height of the flock, since flocks are flat [4], [26]. We measure the shape of the flock by the aspect ratios of the longest relative to the shortest dimension 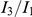 and of the longest over the medium one 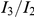. The last ratio 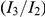, we use as an indication of the degree to which the shape of the flock is oblong independent of the movement direction. We measure the degree to which flocks are elongated in the movement direction as the quotient of its length divided by its width [27], [28]. For this, we enclose the flock in a bounding box parallel to the direction of movement of the centre of gravity of the flock.

The orientation of the shape of the flock is measured by the angle between its longest dimension 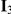 and the direction of the velocity of its centre of gravity 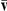. If the angle is acute, the flock is oblong (or elongated) in its movement direction, otherwise the flock is wide (or broad).

The volume of a flock is measured by mapping the position of the individuals on a cubic lattice and counting the occupied lattice cells, the so-called voxelisation method. Here, the cell size is set at the average distance to the nearest neighbours in the flock.

The correlation length of the deviation of the velocity from the average velocity among group members reflects the size of the domains or subgroups of individuals that are closely coordinating and have similar deviations in their velocity. We calculate the correlation length in three steps as was done for real starlings [40]. First, the deviation 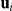 of the velocity of each group member 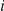 of that of the centre of gravity is calculated (Equ. 23)
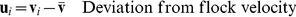
(23)Here, 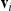 is the velocity of individual 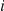, 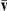 is the velocity of the centre of gravity. Further, the correlation function of the deviations of velocity among all individuals 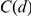 measures the average inner product of the deviations of velocity between individuals at distance 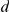 (Equ. 24):
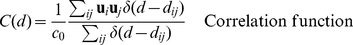
(24)Here, 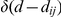 is a smoothed Dirac 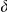-function, 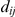 is the distance between two birds and 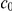 is a scaling factor such that 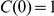. High values of 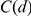 indicate strong correlations in velocity among all flock members at a certain distance. As is typical in flocks the values of the correlation are greater for short distances and become negative for large distances. The correlation length 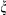 is the distance among birds for which the correlation function is zero. This value reflects the average size of the correlated domains (i.e. the size of the coordinating subgroups).

Polarization is measured globally and locally. Polarization is measured as:
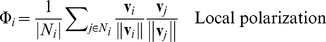
(25)

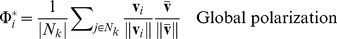
(26)Here, 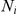 is the set of individuals in the local neighborhood of individual 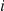 (Equ. 4), 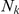 is the set of 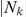 individuals in the flock, 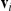 is the velocity of individual 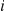 and 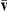 is the velocity of the flock, i.e. the average velocity of its members. Since polarization is based on the dot product of unit vectors it ranges between 0 and 1. Higher values indicate stronger polarization, i.e. higher alignment in the flock.

The average degree of banking is the average over all flock members of the angle between the wings and the horizontal plane.

## Results

**Figure 6:**
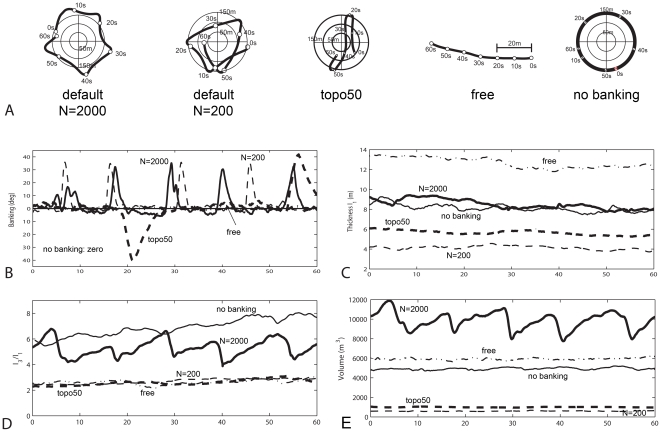
Timeseries. Variability of shape over time (60 s). A) trajectories of a flock of 2000 individuals and of 200 individuals above the roost (default parameters), of a flock of 2000 individuals in which individuals are attracted to 50 nearest neighbours (topo50), a flock of 2000 individuals in which individuals are flying without attraction to the roost (free), and a flock of 2000 individuals in which individuals are flying without banking (nobanking). B) Banking behavior over time. C) thickness, *I_1_*. D) Longest over shortest dimension, the height, 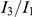. E) Volume.

In the model sharp turns in the trajectory of a flock arise because individuals that are outside the sleeping site are attracted back to it (Fig. 3, Fig. 6A). The turning involves banking (Fig. 6B) and whereas this hardly affects the thickness of the flock, 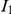 (Fig. 6C), it strongly distorts the aspect ratio, particularly of the longest over the shortest dimension, 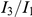 (Fig. 6D) and the volume of the flock (Fig. 6E) (and the average distance to the nearest neighbour; data available on request).

In line with our hypothesis that variability of shape increases when individuals in a flock are less synchronised, higher variability of shape occurs a) when flock size is larger, b) when the number of interaction partners is smaller, c) when the flock turns more strongly, d) when individuals roll into a turn versus when they do not, but, in contrast to our hypothesis, variability of shape decreases when the variability of speed is higher.

**Figure 7:**
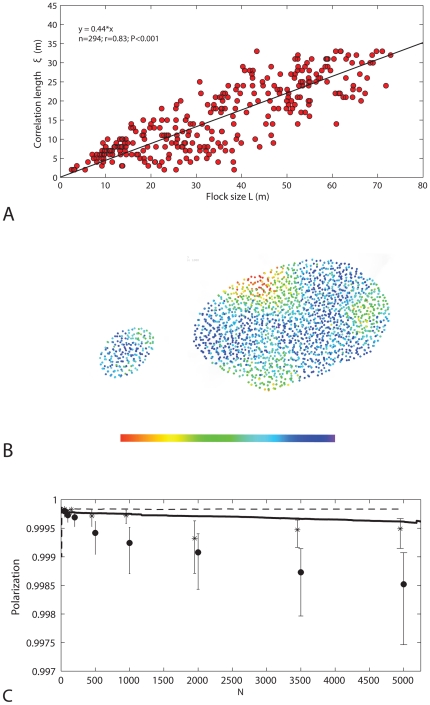
Deviation from average heading and velocity. Deviation from the average of heading and velocity among individuals in the flock. A) Scale free correlation between correlation length of deviation of velocity from that of the centre of gravity versus length of the flock for default values (flock length is measured by the largest distance (in m) between two individuals in the flock); B) Corresponding snapshots of flocks true to scale. From left to right: N = 200 and L≈20 m, N = 2000 and L≈50 m. C) Polarisation (global and local) versus number of individuals in the flock for default parameters and high number of interaction partners (i.e. 50), N = flock size, lines (continuous and striped) indicate local polarization, points indicate global polarization (fat dots: default parameters, stars: 50 interaction partners).

The shape of large flocks is more variable than that of small ones, because individuals at different locations in the flock are more likely to differ in velocity (Movie S2). In larger groups larger sub-flocks form, as reflected in the longer correlation length of the deviation of the velocity from the average (i.e. a scale free correlation, Fig. 7AB). These sub-flocks are not only larger but also more diverse in their movement direction. This becomes clear from the decrease of the global polarisation with flock size, while the local polarisation remains the same (Fig. 7C). Consequently, the variability of behaviour is higher in larger flocks. Besides, in large flocks some individuals bank to return to the roost sooner than others, and consequently turn and lose height earlier than others (Movie S3). By subsequently meeting those above the roost that are still flying outwards, the flock changes shape (aspect ratio) and volume (Fig. 6DE). When the total flock is smaller (N = 200 instead of N = 2000), the shape is more static (Fig. 6DE, movie S4) because a) interactions are more global and b) individuals experience more often the same environment (above or outside the roost).

Similarly, when they have fewer interaction partners, different flock members are more prone to behave differently, because they react to different local environments (Movie S3). When they have more interaction partners synchronisation is stronger which can be seen from two facts (Movie S5). First, the sub-flocks are larger, which is apparent from the stronger increase of the correlation length with the flock size when individuals interact with more neighbours (i.e. with 50 neighbours the slope of the regression line is 0.79 whereas with 6.5 interacting neighbours it is 0.44) and second, their movement direction is less diverse. This is apparent from the stronger local and global polarisation (Fig. 7C, Global polarisation: Wilcoxon matched-pairs signed ranks test, N = 6, Tau = 0, P<0.03 two-tailed; Local polarisation, Mann-Whitney U test, N = 200, z = 9.98, P<0.0001***). Thus, the number of sub-flocks is lower, and their diversity of movement direction is lower and therefore the variability of shape of the flock is less than when there are fewer interaction partners (Fig. 6DE, movie S5 vs S3).

Strong turning happens when individuals fly outside the roost. In large flocks with few interaction partners strong turning induces more variability of shape than moving above the roost with only mild turns (Movie S6). Strong turning, compression of volume of the flock and changes in altitude [26] happen only if individuals roll into the turn. The changes in altitude are a consequence of a temporary reduction of effective lift at the cost of the generation of a centripetal force. If individuals cannot roll, they only turn mildly, remain at the same altitude and the shape of the flock is oblong and continuously bends along the outer edge of the sleeping site (Movie S7). This shape does not resemble real flocks of starlings, because it lacks vertical movement and it is static.

**Figure 8:**
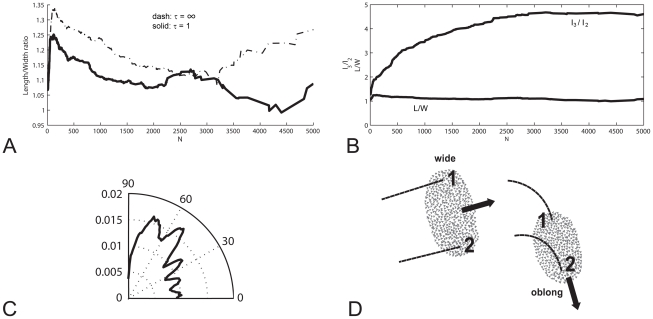
Flock shape and orientation. A) High and low variability of speed (respectively tau = ∞, tau = 1), flock size and degree to which the flock is oblong. B) Oblong in any direction 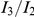 and oblong in movement direction 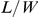 versus number of individuals in the flock. C) Distribution of angles between the movement direction of the flock and its longest dimension 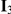 for flock size of 2000 individuals, D) The turning of a flock (view from above). Flock shape changes relative to the movement direction (from wide to oblong), individuals 1 and 2 follow paths of the same length and their location changes in the flock.

Even if we completely omit the force that causes individuals to return to cruise speed (equation 1) it appears possible to increase the variability of speed only marginally from a coefficient of variation of 0.01 to 0.015. This is probably a consequence of the stabilising effect of aerodynamic forces. This increase in speed variability is too small to result in qualitative differences in variability of shape when making sharp turns over the roost. However, when flock members are turning only mildly while flying above the roost, even this small increase of the variability of speed causes flocks of almost all group sizes to become more oblong in the movement direction than at lower variability of speed (Fig. 8A).

Besides, at a low variability of speed the shape of flocks of different sizes appears to be more oblong in other directions than in the movement direction (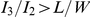, Fig. 8B). The angle between the movement direction and the longest dimension 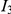 appears to be diverse (Fig. 8C). This diversity of angles is a consequence of the low adjustment of speed, which during turns makes different individuals follow a path of equal length and curvature. Thus they change their movement direction relative to the shape of the flock. This automatically implies that the flock changes its shape relative to the movement direction, e.g. before a sharp turn of 90 degrees, the flock shape is wide and after the turn, it is oblong (Fig. 8D) and it implies that they swap their location in the flock (e.g. before the turn, individual 1 is located at the left, after the turn it is located at the rear).

**Figure 9:**
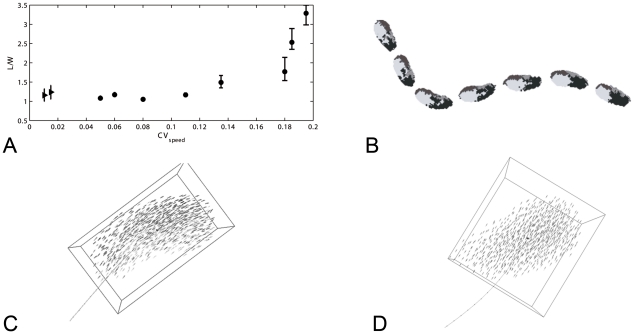
Fish schools. Shape of fish schools [24]. A) Elongation (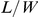) and coefficient of variation of speed in fish model and Stardisplay. Circles: fish school, Triangles: StarDisplay. B) Series of snapshots (with fixed time interval) of a school of 600 individuals indicating the initial location of individuals (at right side at front, at front left side, right side at back, left at back) by four grey-colours. C) Snapshot of school during turning in fish model with extremely high variability of speed (tau = 0.4). Individuals in inner corner automatically slow down and in the outer corner they speed up. Darker grey indicates faster movement. The school is oblong. D) Snapshot of school during turning in fish model with extremely low variability of speed (Tau = 0.02). The school is no longer oblong, but approximately as long as wide. For a color version of Fig. 9BCD, see Supplementary material, respectively, Figs. S1, S2, S3.

Because we can increase variability of speed over a larger range in our fish model [24] than in our bird model, we verify effects of adjustment of speed in our fish model. Upon increasing the parameter tau for the adjustment of speed from 0.05 to 0.34 (Equ. 1) the coefficient of variance of speed increases over a larger range than in our bird model, from 0.05 till 0.20 (whereas in the bird model from 0.01 to 0.015). This results in an increase of the elongation of the shape of the school in the movement direction from being about 1.1 as long as wide to almost 3.5 times (Fig. 9A). Interestingly, in the fish model at parameters, where the coefficient of variance of speed is relatively high, even during turning the shape of the school remains oblong (Fig. 9B; for colour version, Supplementary material, figure S1). This arises, because individuals in the inner corner automatically slow down to avoid collisions and in the outer corner they speed up to remain close to others (Fig. 9C, 2). Consequently, when turning, individuals stick to approximately the same location in the school (indicated in different grey shades, Fig. 9B, S2). At extreme low variability of speed, like in our bird model, during turning school-shape changes its orientation and individuals swap position (Fig. 9D, S3).

## Discussion

We show that local variability of behaviour in a group generally leads to more variable flock-shape, but not in cases of local variability of speed. Instead, high variability of speed results in an oblong shape that is permanently oriented in the movement direction. Remarkably, a lower variability of speed, thus, a stronger synchronisation in a flock, leads to a variable orientation of the longest dimension of the shape relative to the movement direction.

The present study shows that group size has a great impact on the variability of shape. The local variation in larger flocks is greater as is apparent from the greater changes in volume during sharp turns, from the lower global polarisation, and from the scale free correlation of the deviation from the average velocity with flock size in the model. The scale free correlation resembles that in real starlings [40]. The increase in the size of the subgroups with flock size is however larger in our model than in real starlings (gradient of the scale free correlation in the model is 0.44 and in starlings 0.35) indicating that in the model there is less local variation than in reality. This may arise from the greater uniformity of the environment in the model, due to the absence of all kinds of disturbances (such as other birds, including predators, wind, airplanes and very high buildings) [26]. The greater uniformity of environment may also be the cause that the volume of the flock in the model is smaller than in reality [26].

A higher number of interaction partners in our model decreases the variability of flock shape as a consequence of the greater synchronisation of the flock-members (as is apparent from the stronger scale free correlation between subgroup size and flock size, from the stronger global and local polarisation and the smaller changes of volume during turns). Similarly, when in a model of predation on fish schools prey- individuals interact with more neighbours while evading attacks of a predator, the shape of their schools becomes less diverse than when interacting with fewer partners [15].

Turning has a big impact on the variability of shape. Turning in the model resembles descriptions of turning of real flocks, for instance, of rock doves in several aspects [8]. This concerns the temporary changes of volume of the flock and its loss of altitude during a turn, see Fig. 6C of our earlier work [26], the frequently occurring change in orientation of the flock and the repositioning of individuals as shown by Pomeroy and Heppner for rock doves in their Fig. 4B and 5 [8]. Large changes in volume arise only when flocks are large and individuals interact with few neighbours, because in this case individuals sometimes experience different environments (above and outside the roost), which desynchronises behaviour in the flock. Rolling into a turn is essential for creating both the reduction in volume and the loss in altitude in our model. The loss of altitude is a consequence of the reduced lift that individuals experience when banking. Without banking, the shape of the bird flock resembles that of a fish school, since it is very elongated in the movement direction (Movie S7). Together these traits (large flock size, few interaction neighbours and rolling into a turn) cause the great variability of shape.

Change of shape during turning and repositioning of individuals are a consequence of low variability of speed. Repositioning has been observed in several species, such as dunlins [5], pewits [1], rock doves [8] and starlings [4]. Repositioning of individuals in the flock arises, because all individuals follow an equal path length during a turn, as show for rock doves [8]. Low variability of speed causes the change of orientation of the flock and the repositioning of individuals, as is shown in our fish model, because these traits are absent when variability of speed is high (Fig. 9). Here, when adjustability is high, due to the close proximity in the inner corner of the turn individuals slow down and in the outer corner, due to the large inter-individual distances, they speed up. Consequently, during a turn the shape of the school is maintained and individuals stay at approximately the same location in the school. This can be seen in Fig. 9B in which we gave individuals different grey-shades depending on their location in the group in the initial snapshot: they appear to be faithful to approximately the same location, left, front *etcetera* during the whole series of snapshots (for colour-version see Supplementary material, S1). The permanency of shape during turning due to high variability of speed extends our former theory about the causation of the oblong shape of fish schools to include turning behaviour [25]. This theory implies that the group shape becomes more oblong due to frequent slowing down by its members in order to avoid collisions [24], [25], [27], [28]. Our finding that in StarDisplay variability of speed is accompanied also by elongation of the flock in the movement direction, suggests that if their speed could deviate from cruise speed more, this mechanism of elongation would work for birds also. Since shape of fish-schools is more oblong than that of bird flocks, we hypothesise that the variability of speed of birds is lower than that of fish.

There may be several biological advantages to locality of interaction and low variability of speed. Locality of interaction may result in greater variability of behaviour among individuals in a flock. This may confuse predators and reduce their success at catching of prey. Low variability of speed, may confuse predators also through the accompanying repositioning of flock members during turns, the so-called ‘crossing paths’ [8], [41]. Further, it may be advantageous by saving of energy through elimination of acceleration and for avoiding collisions by preventing collisions from front to back [26] (Hemelrijk & Hildenbrandt in prep). Collision avoidance may be more important for birds than for fish, because collisions are more dangerous for birds, because their movement is faster and the viscosity of their medium is lower.

Despite its usefulness, our model has shortcomings. First, of such complex animals as birds, it concerns merely their movement behaviour in relation to the position and heading of others and of the roost, while using a simple model of flying behaviour, ignoring e.g. flapping flight. It ignores any behaviour related to other motivations, such as nutritional [42], reproductive [23] or motivations to avoid a predator [15]. It also ignores environmental disturbances, e.g. by physical forces, such as wind. Thus, in nature, there will definitively be additional reasons that cause flock shape to be variable beyond those that we consider in this paper. Indeed, in the model the variability of shape of, for instance, small flocks of 200 birds is below that observed in real flocks in nature.

A number of the explanations generated by our model can be used as testable hypotheses for empirical data, not only of birds but also of other animals moving in groups. Testable hypotheses from the present investigation concern effects of locality of interaction and variability of speed (Table 2). Particularly in the light of the great effort and difficulties of collecting empirical data of three dimensional positions of animal groups [25] and particularly of flocks of birds [6], [16], such model-based hypotheses are valuable.

**Table 2:** Hypotheses for empirical testing derived from the model.

1) Greater locality of interaction causes more variable shape in terms of volume and aspect ratios
a) in larger groups
b) when individuals interact with relatively fewer interacting partners
c) in a heterogeneous environment
2) Lower variability of speed causes higher variability of shape
a) It induces shape to be less oblong in the movement direction
b) It induces an almost random orientation of the oblong shape
c) It causes changes in the orientation of the shape relative to the movement direction during turning
d) It causes individuals to reposition themselves in the group during turns
3) Higher variability of speed is expected
a) in fish rather than in birds
b) to result in slowing down in inner corners during turning

## Supporting Information

Figure S1 (TIF)

Figure S2 (TIF)

Figure S3 (TIF)

Movie S1 **Measurement of school shape.** This movie shows a bounding box around the flock in black. Its dimensions are calculated with the PCA. The the shortest dimension is the height. The flock is clearly asymmetrical or oblong. Simultaneously the movie shows the bounding box for measuring the degree to which the flock is elongated in the movement direction (white).
(WMV)

Movie S2 **Deviation of global velocity.** This movie shows how clusters of coordinating individuals with similar deviation of velocity come and go. Blue indicates no deviation from velocity of center of gravity, red indicates maximal deviation.
(WMV)

Movie S3 **A turning flock of 2000 individuals.** This movie shows a flock of 2000 individuals under default parameters above the sleeping site. The shape compresses and changes when the flock turns at the border of the sleeping site.
(WMV)

Movie S4 **A turning flock of 200 individuals.** This movie shows a flock of 200 individuals under default parameters above the sleeping site the shape hardly changes when the flock turns at the border of the sleeping site.
(WMV)

Movie S5 **A turning flock with individuals interacting with 50 interaction partners.** This movie shows a flock of 2000 individuals in which the individuals interact with their 50 closest neighbours. Consequently, the volume is small, the distance to the nearest neighbours is short and the shape is constant.
(WMV)

Movie S6 **Flying above roost with mild turns.** A flock of 2000 individuals (at default parameters) moves approximately straightforward. The shape hardly changes.
(WMV)

Movie S7 **Without banking.** This movie shows a flock of 2000 individuals in which the individuals do not bank while turning. Consequently, a flock emerges that is oblong and moves along the circular border of the sleeping site.
(WMV)
