# Supplementary figures and images for: Some Causes of the Variable Shape of Flocks of Birds
Source: PLoS One. 2011 Aug 4;6(8):e22479. doi: 10.1371/journal.pone.0022479 (PMC3150374; doi:10.1371/journal.pone.0022479)

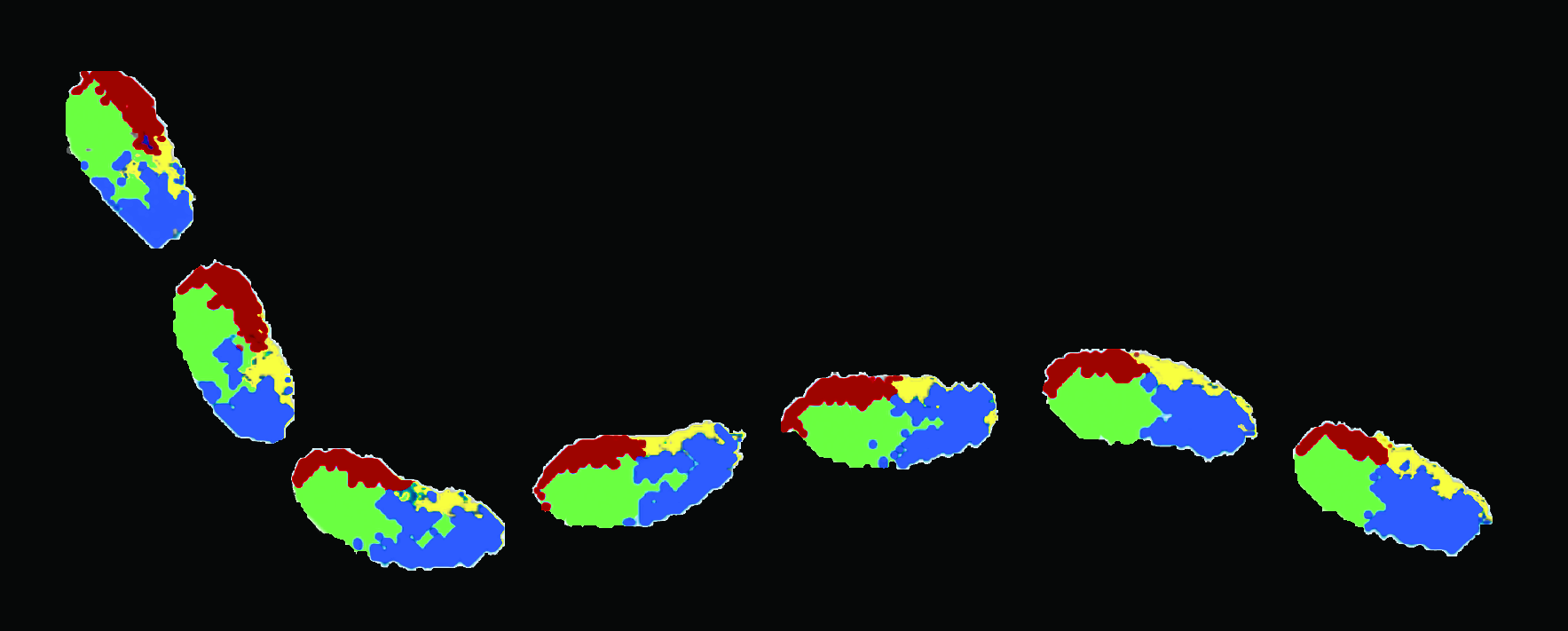

Supplement: Figure S1 — (TIF) [file pone.0022479.s001.tif]

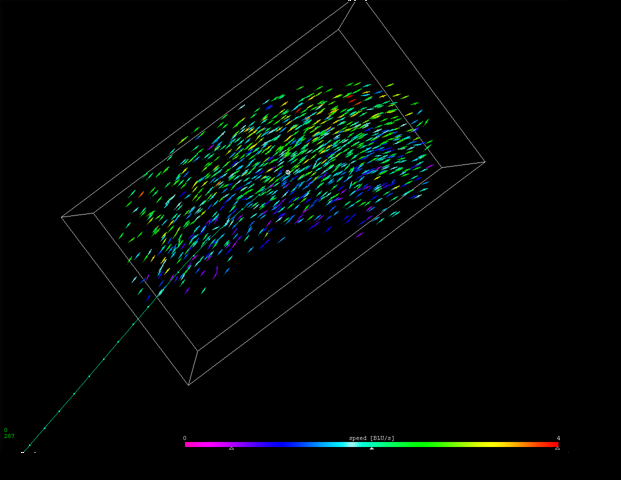

Supplement: Figure S2 — (TIF) [file pone.0022479.s002.tif]

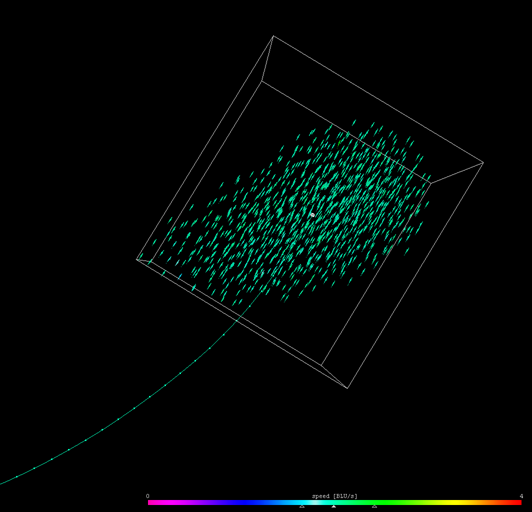

Supplement: Figure S3 — (TIF) [file pone.0022479.s003.tif]
